# Prevalence and associated risk factors for Kaposi’s sarcoma among HIV-positive patients in a referral hospital in Northern Tanzania: a retrospective hospital-based study

**DOI:** 10.1186/s12885-018-5155-2

**Published:** 2018-12-17

**Authors:** George P. Semango, Renard M. Charles, Consolata I. Swai, Alex Mremi, Patrick Amsi, Tolbert Sonda, Elichilia R. Shao, Daudi R. Mavura, Leo A. B. Joosten, Elingarami Sauli, Mramba Nyindo

**Affiliations:** 1 Kilimanjaro Christian Medical University College, P.O Box 2236, Moshi, Tanzania; 2 Regional Dermatology Training Centre, Kilimanjaro Christian Medical Centre, P.O. Box 8332, Moshi, Tanzania; 3 Department of Medicine, Kilimanjaro Christian Medical Centre, P.O. Box 3010, Moshi, Tanzania; 4 Department of Pathology, Kilimanjaro Christian Medical Centre, P.O. Box 3010, Moshi, Tanzania; 5 Department of Internal Medicine, Radboud Center for Infectious diseases (RCI), Radboud University Medical Center, P.O. Box 9101, 6500 HB Nijmegen, The Netherlands; 6 Radboud Center for Infectious diseases (RCI), Radboud University Medical Center, P.O. Box 9101, 6500 HB Nijmegen, The Netherlands; 7 School of Life Sciences and Bioengineering, Nelson Mandela-African Institution of Science and Technology, P.O. Box 447, Arusha, Tanzania

**Keywords:** HIV-related Kaposi’s sarcoma, Prevalence, Risk factors, Northern Tanzania

## Abstract

**Background:**

Kaposi’s sarcoma (KS) is a multifocal angioproliferative tumor involving blood and lymphatic vessels, caused by Human Herpes Virus-8 (HHV-8). KS is an important AIDS-defining tumor with high prevalence in Sub-Saharan Africa, including Tanzania which has high HIV and HHV-8 sero-prevalence. It is critically important to monitor the prevalence of AIDS-defining tumors, such as KS, in the age of HIV/AIDS. We studied the prevalence of KS and associated risk factors among HIV-positive patients at Kilimanjaro Christian Medical Centre (KCMC), a referral hospital in northern Tanzania, over the period from January 2012 to December 2015.

**Methods:**

This was a retrospective hospital-based cross-sectional study to determine the prevalence of KS among HIV/AIDS patients between 2012 and 2015. The study included 1100 HIV patients’ data which were collected at the Infectious Disease Clinic (IDC) from patients’ files. Stata version 13 (StataCorp LP, Texas 77,845 USA) was used for all statistical analyses. The prevalence of KS was calculated across levels of a number of categorical variables. Logistic regression was performed to determine relative risk of KS for all characteristics. We included all variables with *p*-values ≤10% in the multivariate analysis, including ART use, as this is considered to have an influence on KS. In the multivariate analysis, statistical significance was established based on a two-tailed p-value ≤5%. All patients’ notes were kept confidential as per the Helsinki declaration.

**Results:**

Our results revealed a 4.6% prevalence of KS at KCMC hospital, between January 2012 and December 2015, 51(4.6%) patients were diagnosed with KS out of 1100 HIV-positive patients. The study further revealed that KS in HIV patients was most associated with low CD4 cell count (less than or equal to 200 cells/μl). Moreover, women were more likely than men to diagnosed with KS, with higher odds significantly associated with KS (OR 0.42, *p* < 0.009). Increased age, above 35 years, among the HIV seropositive patients was significantly associated with KS (OR 25.67, *p* < 0.007). HIV patients who were none smokers were more likely to suffer from KS compared to HIV smokers (OR 0.41, *p* < 0.010).

**Conclusion:**

KS remains a common malignant vascular tumor commonly associated with HIV/AIDS in Tanzania. Our study highlights the need for continued efforts to combat HIV, as well as associated diseases such as KS. Continued availability of ART (Anti-Retroviral Therapy) to HIV/AIDS patients, and test reagents for CD4 cell count and viral load determination are important measures to alleviate the suffering of these patients. Furthermore, studies to gather more evidence on ART resistance are highly needed to guide treatment choices.

## Background

Kaposi’s sarcoma (KS) was first described in 1872 by Moritz Kaposi, a Hungarian dermatologist, who described KS as a rare multifocal angioproliferative tumour involving blood and lymphatic vessels, occurring only in Eastern Europe and the Mediterranean [1]. KS is a cancer that causes lesions to grow in the mucosa such as skin, mucous membranes lining the mouth, nose, throat or viscerally in lymph nodes, or other internal organs [1, 2]. KS presents as either red, brown or purple lesions that can be typed into different clinicopathological forms, such as patch, plaque, nodular, lymphadenopathic or infiltrative ulcers. It can also present as florid severe swellings in the arms, legs, face or scrotum. The etiological agent associated with KS was discovered in 1994 and is called Human Herpes Virus 8 (HHV8), also known as KS-associated Human Herpes Virus (KSHV) [1, 3]. There are 4 classes of KS, which include; Classic KS, Endemic KS, Immunosuppression-associated KS and AIDS-associated KS [4]. AIDS-associated KS is the most prevalent form of KS, and its prevalence tremendously increased post-HIV era but later declined due to introduction of Anti-Retroviral Therapy (ART). However, KS still remains one of the leading cancers in HIV-infected individuals, especially in sub-Saharan Africa, including Tanzania [5, 6]. Uldrick et al., reported post-HIV seroprevalence of KSHV as very common in sub-Saharan Africa, with seropositivity rates above 50%; moderately prevalent in Mediterranean countries (20–30%), but much less common (< 10%) in most of Europe, Asia and the US [7]. In Tanzania, the prevalence of AIDS-related KS was reported to be 2.4% at Bugando medical center in northwestern Tanzania from 2004 to 2014 [8], whereas Koski et al.*,* reported prevalence of AIDS-associated KS to have dropped from 10.1% in 2003 to 7.4% in 2011 in a study conducted at Ocean Road Cancer Institute (ORCI), Tanzania [9]. A study conducted from 2006 to 2007 at The Kilimanjaro Christian Medical Centre (KCMC) Regional Dermatology Training Center (RDTC) and Mawenzi Regional Hospital Infectious Diseases Clinic in Moshi, northern Tanzania, reported a 4% prevalence of KS [10]. These studies show that KS is still prevalent in Tanzania. Furthermore, according to the International Agency for Research on Cancer 2012 report, KS accounts for approximately 12.3% of all cancer deaths in the East African region [6].

Several risk factors have been associated with high prevalence of KS in sub-Saharan Africa and East Africa. The major risk factors include HIV seropositivity and non-adherence to anti-retroviral therapy [9], as well as low CD4 cell levels [3, 11, 12]. In contrast, injecting drug users and homosexuals are the highest risk groups for KS in developed countries, such as the United States of America [11], where it is estimated that 30–40% of homosexual men infected with HIV are seropositive for HHV-8 [11]. Similar observations were made in India, where Munawwar et al., reported the major risk factor for KS among HIV patients to be seropositivity to HHV-8, which accounted for 26.0% in heterosexual men and 25% in men who had sex with other men (MSM) developed KS [13].

Treatment options for KS include surgical excision, radiation therapy and intralesional chemotherapy. These treatment options are used based on disease severity and available local treatment options. Highly Active Anti-Retroviral Therapy (HAART) is recommended to reduce the extent and size of KS lesions in HIV-related KS patients. However, recent reports have shown that ART resistance is on the rise in southern and eastern Africa and Latin America and, as a result, it may soon be necessary to change the recommended first-line antiretroviral drug regimen in many countries to integrase inhibitor-based treatment [14].

Generally, KS continues to be one of the leading AIDS-defining illnesses in Sub-Saharan Africa, including Tanzania, as well as one of the most prevalent cancers overall due to HIV and HHV-8 [13, 15]. In light of its importance in the HIV era, KS is thus a critical tumor to monitor on an annual and periodic basis, to assess the impact of different preventive and management strategies in place against the disease. The aim of this study was therefore to determine the prevalence of KS and associated risk factors among HIV positive patients who attended KCMC referral hospital in Kilimanjaro between 2012 and 2015.

## Methods

### Study design

This study was a hospital based retrospective cross sectional study to determine prevalence of KS and associated risk factors among HIV positive patients who attended KCMC referral hospital in northern Tanzania. The study involved collection and analysis of secondary data from patients’ records from 1 January 2012 to 31 December, 2015.

### Study area

This study was conducted at KCMC, a tertiary referral hospital in Kilimanjaro region, northern Tanzania. Kilimanjaro region has 7 districts; Moshi municipality where the hospital is found, Moshi rural, Same, Rombo, Mwanga, Hai and Siha. The hospital hosts the Infectious Disease Clinic (IDC) and Regional Dermatology Training Centre (RDTC) that are routinely attended by KS patients. The hospital serves not only the Kilimanjaro region population, but also the wider population of northern Tanzania, estimated to be around 15 million people. The hospital also attends patients referred from various hospitals in Tanzania, with 500–800 outpatients per day, and 630 official beds.

### Study population

All data for HIV-positive patients who attended the IDC at KCMC from 1 January 2012 to 31 December 2015 were included in this study. The data included patients’ HIV serostatus, CD4 cell count and ART adherence. The data was collected at admission during the study period. Prevalence of KS was calculated based on HIV-positive patients diagnosed with KS in the study period. All included patients were 18 years old and above. KS patients were first diagnosed clinically by physical examination by a dermatologist, followed by histological examination of punch biopsies from suspected lesions and finally confirmed by immunohistochemistry.

### Sampling technique

Data was collected by reviewing patients’ medical records available at KCMC IDC. It included all HIV-positive patients diagnosed with KS from 1 January 2012 to 31 December 2015.

## Data collection method and tools

### Data collection method

Data was collected by retrospective medical records review of HIV patients’ files available at the KCMC medical records unit. Data on serostatus was collected first and socio-demographic and other related data were collected thereafter. Data included CD4+ cell count at admission, history of sexually transmitted diseases (STD), history of being attended by a traditional healer, ART use, cigarette smoking, marital status, education, residence and occupation. These data were collected from the first visit of the patient. All included HIV patients with KS must have been reviewed by a dermatology specialist clinically and histologically confirmed by routine haematoxylin and eosin staining and analyzed by two independent, trained dermatopathologists. Anti-LANA immunohistochemistry was finally done to confirm the histology diagnoses. The immunohistochemistry was also used as the final confirmation where the diagnosis was indeterminate by routine histology.

### Statistical data processing and analysis

Data collection sheets were checked first for completeness and accuracy. Data entry was done using MS Excel 2013; Stata v13 was used for all analyses. Categorical variables were analyzed using Chi-square tests. T-test was used to compare mean age of participants between KS+ and KS-. Logistic regression analysis was used to identify risk factors and calculate risk magnitudes by Odds Ratio (OR). Factors included were age, gender, marital status, occupation, smoking, visit to traditional healer, CD4 cell counts, and STI history. Age was stratified into 3 strata of 0 to 18, 19 to 35 and 35 and above based on categories of young, young adults and older age as per Tanzanian life expectancy. CD4 count was stratified in stata of 0–200, 201 to 600 and 600 and above based on the WHO categorization of 0–200 being considered AIDS, 201 to 600 intermediate and 601 and above is considered to be within normal range. ‘Student’ was used as the reference group for occupation so as to be consistent with ‘age’ as students are generally expected to be in the young individuals strata. Univariate and multivariate analyses were used to determine unadjusted and adjusted OR respectively. Statistical significance was established using a two-tailed *p*-value ≤5% in all analyses. We included all variables with *p*-values ≤10% in the multivariate analysis, including ART use, as this is considered to have an influence on KS. The variables included in the multivariable analysis were age, gender, marital status, occupation, smoking, traditional healers,ART use, CD4 count and history of STIs. In the multivariate analysis, statistical significance was established based on a two-tailed p-value ≤5%.

## Results

### Socio- demographic characteristics of the study participants

**Fig. 1:**
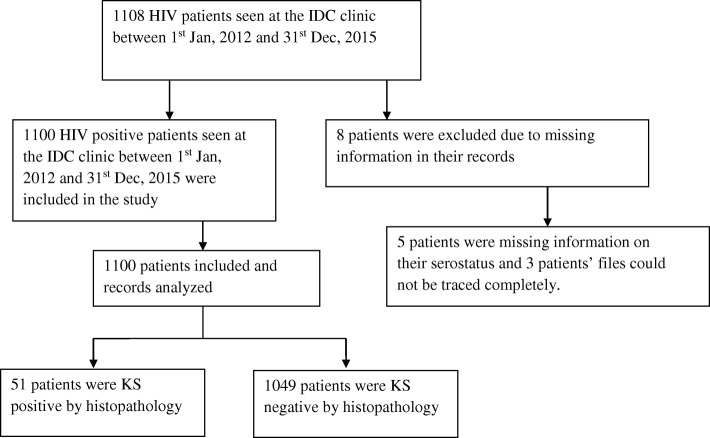
Flow diagram showing the number of patients included and excluded in the study conducted. A flow diagram showing the number of patients’ data included in this study. The diagram summarizes the patients found to be positive for KS among all the patients that tested positive for HIV during the study period

A total of 1108 HIV-positive patients’ data was retrieved for the period from 1 January 2012 to 31 December 2015. Eight patients were excluded due to lack of vital information on serostatus and other records. A total number of 1100 HIV patients’ data were thus used in this study, as summarized in Fig. 1 below. Sixty-one percent of the study participants were females and 39% were males. Approximately 61.5% had primary school education level. About 49.6% of the study participants were married, 56.2% resided in rural areas, and 35.7% were peasants. The socio-demographic data is summarized in Table 1 below.

**Table 1:** Socio-demographic and prevalence of KS in different characteristics of patients seen at IDC clinic between January 2012 and December 2015

Characteristic		Kaposi sarcoma	
	Total (%)	Negative (%)	Positive (%)
Age (years)			
0–18	189(17.2)	188(17.9)	1(2)
19–34	481(43.7)	463(44.1)	18(35.3)
35+	430(39.1)	398(37.9)	32(62.7)
Gender			
Male	429(39)	398(37.9)	31(60.8)
Female	671(61)	651(62.1)	20(39.2)
Marital status			
Married	546(49.6)	512(48.8)	34(66.7)
Divorced(widowed	212(19.3)	206(19.6)	6(11.8)
Single	342(31.1)	331(31.6)	11(21.6)
Residence			
Urban	482(43.8)	464(44.2)	18(35.3)
Rural	618(56.2)	585(55.8)	33(64.7)
Occupation			
Student	275(25)	270(25.7)	5(9.8)
Employee	150(13.6)	139(13.3)	11(21.6)
Business	282(25.6)	272(25.9)	10(19.6)
Peasant	393(35.7)	368(35.1)	25(49)
Education level			
No formal education	95(8.6)	87(8.3)	8(15.7)
Primary	677(61.5)	650(62)	27(52.9)
Secondary	241(21.9)	233(22.2)	8(15.7)
College	87(7.9)	79(7.5)	8(15.7)
Smoking			
Smoker	241(21.9)	216(20.6)	25(49)
Non smoker	859(78.1)	833(79.4)	26(51)
Visit to traditional healers			
No	913(83)	885(84.4)	28(54.9)
Yes	187(17)	164(15.6)	23(45.1)
ART use			
ART user	807(73.4)	767(73.1)	40(78.4)
Non ART user	293(26.6)	282(26.9)	11(21.6)
CD4 count			
0–200	405(36.8)	364(34.7)	41(80.4)
201–600	531(48.3)	522(49.8)	9(17.6)
601+	164(14.9)	163(15.5)	1(2)
History of STIs			
Have STI	208(18.9)	192(18.3)	16(31.4)
No STI	892(81.1)	857(81.7)	35(68.6)

The table summarizes the socio-demographic and prevalence characteristics of the 1100 patients included in this study. The general characteristics of the study population are summarized. Furthermore, we observed more KS positivity in older patients above 35 years. More male patients were also KS positive compared to female HIV positive patients. Other patients’ characteristics which were more common on KS patients were smoking, attending traditional healers and low CD4+ counts

### Prevalence and distribution of KS among HIV seropositive patients by socio-demographic characteristics

Out of 1100 HIV patients, 51 (4.6%) patients were histologically confirmed KS positive. KS was most prominent in the 35+ age group. Females were significantly more likely to present with KS (OR 0.42, *p* = 0.009) than males. KS patients with CD4 cell count of less than or equal to 200 cells/μl were more likely to have a KS diagnosis compared to patients with higher CD4 cell counts of 201 and above (OR 28.46, *p* < 0.003). Table 2 summarizes the data.

**Table 2:** Risk magnitude (unadjusted OR) and Adjusted risk magnitudes (adjusted OR) association of each patient characteristic with KS as calculated in a univariate and multivariate logistic regression analysis model

Characteristic	Univariate regression			Multivariate regression		
	OR^c^	95% CI	Pvalue	OR^a^	95% CI	Pvalue
Age (years)						
0–18	ref			ref		
19–34	7.31	[0.97,55.14]	0.054	10.10	[1.07,95.45]	0.044
35+	15.12	[2.05,111.46]	0.008	25.67	[2.44,270.13]	0.007
Gender						
Male	ref			ref		
Female	0.39	[0.22,0.70]	0.002	0.42	[0.22,0.81]	0.009
Marital status						
Married	ref			ref		
Divorced(widowed	0.44	[0.18,1.06]	0.067	0.44	[0.17,1.16]	0.095
Single	0.50	[0.25,1.00]	0.051	2.34	[0.79,6.95]	0.126
Residence						
Urban	ref					
Rural	1.45	[0.81,2.62]	0.211			
Occupation						
Student	ref			ref		
Employee	4.27	[1.46,12.54]	0.008	2.10	[0.46,9.51]	0.338
Business	1.99	[0.67,5.89]	0.216	0.57	[0.12,2.65]	0.475
Peasant	3.67	[1.39,9.71]	0.009	0.99	[0.20,4.78]	0.986
Education level						
No formal education	ref					
Primary	0.45	[0.20,1.03]	0.058			
Secondary	0.37	[0.14,1.03]	0.056			
College	1.10	[0.39,3.07]	0.854			
Smoking						
Smoker	ref			ref		
Non smoker	0.27	[0.15,0.48]	0.000	0.41	[0.21,0.81]	0.010
Visit to Traditional healers						
No	ref			ref		
Yes	4.43	[2.49,7.89]	0.000	2.99	[1.49,6.00]	0.002
ART use						
ART user	ref			ref		
Non ART user	0.75	[0.38,1.48]	0.403	2.07	[0.79,5.40]	0.137
CD4 counts						
0–200	18.36	[2.50,134.62]	0.004	28.46	[3.11,260.90]	0.003
201–600	2.81	[0.35,22.35]	0.329	3.88	[0.43,35.50]	0.229
600+	ref			ref		
History of STIs						
Have STI	ref			ref		
No STI	0.49	[0.27,0.90]	0.022	0.53	[0.26,1.09]	0.083

This table summarizes the crude and adjusted risk magnitude of KS among the patients’ different characteristics. The table gives the likelihood of acquiring KS among the different patients’ characteristics

OR^a^ adjusted Odds ratios

OR^c^ crude Odds ratios

### Risk factors associated with KS among HIV seropositive patients

Our data showed that more HIV patients with CD4 count less than or equal to 200 cells/μl were more likely to be diagnosed with KS compared to patients with CD4 cell count above 200 cells/μl (OR 28.46, p < 0.003). When we compared the ART users to non-ART users by using adjusted odds ratio, the non-ART users were not more likely to suffer from KS compared to ART users (OR 2.07, *p* < 0.137). Moreover, women were more likely than men to be diagnosed with KS, with higher odds significantly associated with KS (OR 0.42, *p* < 0.009). Increased age among the HIV seropositive patients was significantly associated with KS (OR 25.67, *p* < 0.007).The age distribution in Table 2 shows patients aged over 35 years were more likely than those below or equal to 18 years to present with KS (OR 25.67, p < 0.007). HIV patients who were smokers were not more likely to suffer from KS compared to HIV nonsmokers (OR 0.41, *p* < 0.010). Risk factors associated with KS are summarized in Table 2 below.

## Discussion

We have reported a prevalence of 4.6% KS among HIV positive patients attending KCMC hospital in the 4-year study period from 2012 to 2015. This prevalence is slightly higher than that reported by Mavura et al.*,* 2015 in the same region [10]. Furthermore, similar studies done within the past 5 years in the same area but in different settings, reported lower prevalence of KS. These studies were conducted in South Africa, northwestern Tanzania, and northern Tanzania, and reported prevalences of 3.4, 2.4 and 4% respectively. Other studies conducted in mediterranean countries and within the East African region [6] reported KS prevalence of 10–20, and 12.3%, which is higher than what we have reported herein. These differences may be due to heterogeneity in the distribution of KS within Tanzania as well as elsewhere. This may also be contributed by other factors, including length of the study period, under/over reporting and misdiagnosis.

Our data showed an association between CD4 cell count and KS, where low CD4 cell count (≤200) was associated with increased odds for KS 28.4 fold (*p* = 0.003). Similar observations were made by other studies conducted in Nigeria [16] and northwestern Tanzania [8]. These similar findings strongly suggest that a low CD4 cell count is associated with KS. A possible explanation for this is that KS strongly impacts on immune responses. More studies on immune response mechanisms in KS will possibly help provide explanations for observed differences in prevalence of KS and CD4 cell count.

Furthermore, our findings pointed out that the HIV positive non-ART users were not more associated with KS compared to ART users. Use of ART was not shown to have a protective effect against HIV and thus KS as previously shown by other investigators from different geographical areas [9, 12]. Most patients at the time of this study were eligible for ART if they presented WHO HIV stage IV or with CD4 cell counts < 200 cells/μl. The protective role of ART against KS has been explored extensively by other investigators [13, 18]. ART raises body immunity in HIV infected persons, and this may indirectly benefit KS patients, therefore the interaction between HIV-KS and ART should be further investigated. Early initiation of ART as per newly established WHO standards is also highly encouraged as it may as well be protective against KS. However, there are increasing concerns of ART resistance worldwide and more so in East Africa [14, 17]. In addition to ART resistance, there are policy issues that need to be addressed to help people re-engage in care and reduce loss of patients from care, as these are important factors contributing to the development of drug resistance [18]. ART drug resistance was reported to be around 14.9% in ART-naïve patients in Tanzania in 2008 and has risen to 25.4% in 2016 [19, 20].

Our study findings showed that females were more likely to be diagnosed with KS compared to males (*p* < 0.009). This observation is different from that reported in other studies conducted in Nigeria [21] and Dar-es-salaam [9] by Kagu et al., 2006 and Koski et al., 2015, respectively, as well as by Ferlay et al., who reported an incidence of 5.5 in males against 2.9 in females [5]. Reasons for this disparity are yet to be elucidated. Moreover, a study done in northwestern Tanzania reported AIDS-related KS to be more severe and to progress faster in females. The study did not identify reasons for the disparity, but ruled out association with immunological responses since there was no significant difference between CD4 cell counts in males and females [8].

Our study observed age to be significantly associated with KS. Patients over 35 years were 25.7 times more likely to have KS than the age groups below (*p* < 0.007). Similar findings were reported in studies conducted elsewhere [10]. This may be explained by reduction of immunity as people get older, putting them at increased risk for age-related chronic diseases, including cancer.

Treatment of HIV patients by traditional healers had increased risk for suffering from KS. This may be explained by traditional healers contributing to delaying of ART usage by HIV patients in the process of seeking treatment from the healers, thus increasing their risk for KS. The same finding was observed in Cameroon [22, 23].

Our results generally show that there is still need for more well-defined approaches in curbing KS among the HIV seroconverted patients. Moreover, the ongoing interventions need to be strengthened, especially early initiation of ART regardless of CD4 cell count as recommended by the WHO, because ART has been associated with better prognosis and prolonged life of HIV-positive patients, including lowering of KS seroprevalence when proper adherence is observed. Other interventions include increasing awareness among HIV patients on compliance/adherence to ART; nutritional care and support for people living with HIV/AIDS to improve their immunity and adherence to ART; health care education especially as patients’ age, as well as health seeking attitude. We also propose that monitoring of ART resistance and virological failure, as reported by other investigators, should also be carried out in Tanzania. Although we have not directly studied this, we propose that there could be an association between increased KS prevalence and virological failure, as reported to be an emerging issue in HIV patient care.

### Limitations

Our study had a number of limitations. Difficulty in acquiring patient information from hospital files led to limiting the number of study participants, as those patients who lacked the required information for the study were excluded from the study. Potential confounding factors may have been responsible for differences in the findings. For example, genetic and hormonal differences between males and females may have contributed to the observed differences in the analyzed data. This also warrants further mechanistic studies.

Finally, our study highlights the need for continued efforts in combating HIV/AIDS and its associated KS in Tanzania. Specialized treatment for KS and other cancers should be advocated. This study also highlights the need for regular evaluation of HIV/AIDS interventions and guidelines established by the WHO against the HIV pandemic in different zones and regions of the World, especially in areas with the highest prevalence of HIV/AIDS. We also propose further studies to evaluate the level of ART resistance in the region, to elucidate its association with prevalence of KS in HIV patients. Efforts are undertaken in the coastal region (Dar es Salaam) and south-central part of Tanzania, where virological failure was recently reported to be 14.9 and 25.4% respectively. However, we are not aware of a country-wide approach being put in place yet.

## Conclusion

KS remains a common malignant vascular tumour commonly associated with HIV/AIDS in Tanzania. The prevalence of KS among HIV-positive patients at KCMC referral hospital during 2012–2015 period has been found to be 4.6%, and has remained around 4% in Northern Tanzania. Our study highlights the need for continued efforts in combating HIV, including combating its associated diseases like KS. Continued availability of test reagents for CD4 count and viral load determination, and early initiation of ART to HIV/AIDS patients will alleviate the suffering of these patients. Further studies on ART resistance are highly needed to guide decisions on appropriate HV/AIDS management.

## Abbreviations

IDC: Infectious Disease Clinic
KCMC: Kilimanjaro Christian Medical Centre
KS: Kaposi’s sarcoma
RDTC: Regional Dermatology Training Centre
